# Antimicrobial Effect of Waterborne Polyurethane-Based Cellulose Nanofibril/Silver Nanoparticles Composites and *Acacia concinna* (Willd.) DC Extract (Shikakai)

**DOI:** 10.3390/polym16192683

**Published:** 2024-09-24

**Authors:** Lu Lu Taung Mai, H’ng Paik San, Min Min Aung, Hiroshi Uyama, Ainun Zuriyati Mohamed, Ezyana Kamal Bahrin, Mas Jaffri Masarudin, Azra Afrina binti Mohamad Zulkifli, Tung Woey Chew

**Affiliations:** 1 Higher Education Centre of Excellence (HiCoE), Institute of Tropical Forestry and Forest Products (INTROP), Universiti Putra Malaysia, Serdang 43400, Selangor, Malaysia; gs56760@student.upm.edu.my (L.L.T.M.); ainunzuriyati@upm.edu.my (A.Z.M.); chewtungwoey@gmail.com (T.W.C.); 2 Department of Chemistry, University of Myitkyina, Myitkyina 01011, Kachin State, Myanmar; 3 Department of Forestry and Environment, Faculty of Forestry, Universiti Putra Malaysia, Serdang 43400, Selangor, Malaysia; 4 Department of Applied Chemistry, Graduate School of Engineering, Osaka University, 2-1 Yamadaoka, Suita 565-0871, Osaka, Japan; uyama@chem.eng.osaka-u.ac.jp; 5 Department of Bioprocess Technology, Faculty of Biotechnology and Biomolecular Sciences, Universiti Putra Malaysia, Serdang 43400, Selangor, Malaysia; ezyana@upm.edu.my; 6 Department of Cell and Molecular Biology, Faculty of Biotechnology and Biomolecular Science, Universiti Putra Malaysia, Serdang 43400, Selangor, Malaysia; masjaffri@upm.edu.my; 7 Department of Chemistry, Faculty of Science and Technology, Universiti Putra Malaysia, Serdang 43400, Selangor, Malaysia; azrazulkifli@gmail.com

**Keywords:** *Acacia concinna* (Willd.) DC (AC), cellulose nano fibrils (CNFs), waterborne polyurethane (WPU), silver nanoparticles (AgNPs)

## Abstract

Antimicrobial coatings are becoming increasingly popular in functional material modification and are essential in addressing microbial infection challenges. In this study, the phytochemical and antimicrobial potential of aqueous, 80% methanol and 80% ethanol pod extracts of *Acacia concinna* (Willd.) DC (AC) and its application in the green in situ (one pot) synthesis of silver nanoparticles on Cellulose nano fibrils (CNF) and Waterborne polyurethane (WPU) were prepared. The phytochemical evaluation of *Acacia concinna* crude extracts showed the presence of alkaloids, flavonoids, phenols, tannins, terpenoids, saponins, steroids. The surface plasmon Resonance peak of CNF/AC-AgNPs was 450 nm and the FTIR result confirmed functional groups such as carbonyl, phenols and carboxyl were present which was important for the bio-reduction of silver nanoparticles. The crude AC aqueous pods extract against Gram-positive and Gram-negative bacteria compared with AC ethanol and AC methanol extracts. The WPU/CNF/AC-AgNPs composite dispersion was also good in terms of its antibacterial activities. The WPU/CNF/AC-AgNPs nanocomposites could be applied as bifunctional nanofillers as an antimicrobial agent in food packaging systems and other biological applications.

## 1. Introduction

In research from [1], silver nano-particles (AgNPs) have been shown to be very stable at elevated temperatures and have a high surface-to-volume ratio. Additionally, because of their antibacterial and filling capabilities, AgNPs may be used as food packaging materials. A recent work showed that the inclusion of AgNPs enhanced the tensile strength and water vapour barrier properties of a cellulose-based nanocomposite, while also exhibiting apparent antibacterial activity against *Escherichia coli* and *Staphylococcus aureus*.

Cellulose nanofibrils (CNFs) are a kind of nanocellulose with a fibril width of 10–100 nm and a length of typically more than 1 μm. CNFs may be utilised to enhance the mechanical qualities and active functionalities of food packaging materials due to their distinctive architectures. Nanofibrillated cellulose (NFC) is generally created by mechanical shearing in water, with or without pretreatment, and is a promising new bio-based nanomaterial. Its low density, easy biodegradability and renewal, low cost, low thermal expansion and high strength make it a promising candidate for use in many applications, including nanocomposite films, hydrogels and foams with fibril network structures or a close packing structure [2].

Waterborne polyurethane (WPU) is one of the most ecologically friendly materials in the field of surface coatings and composite materials. Polyurethanes made from vegetable oil have gained popularity as a renewable material [3,4,5]. In this study, jatropha oil is used as a raw material for the manufacturing of polyols and polyurethane due to the presence of unsaturated fatty acids. Waterborne polyurethane is a well-dispersed mixture stabilised by electrostatic repulsive force, rather than being watery [6,7,8]. WPU also has a number of benefits, including great flexibility at low temperatures, being pollution-free and non-inflammable and has good application and non-toxicity [9,10,11]. Generally, antibacterial nanomaterials like silver nanoparticles [12,13,14] and copper nanoparticles [15,16], titanium dioxide nanoparticles [14], quaternary phosphonium salt [15], Gemini quaternary ammonium salt [16], p-hydroxybenzonic anionic intercalated MgAl-layered double hydroxides [17] and 2-aminebenzothiazole [18] have been combined with WPU to create antibacterial WPU. Plant-based antimicrobial agents have certain limitations, despite several papers demonstrating the effective antibacterial capabilities of WPU coated with antibacterial chemicals.

In this study, *Acacia Cocina* (Willd.) DC (AC) is the plant that will be studied as it not only has phytochemical and antimicrobial potential but also acts as a reducing agent in silver nanoparticles synthesis. Situated in Southeast Asia, the *Acacia concinna* tree is known as shikakai. There are many species of Leguminosae that are related to this plant. It is already in use as a shampoo as well as in a variety of medical products. These pods are abundant in saponins, which include flavonoids and monoterpenoids. Saponins are natural crude extracts derived from plants [19,20,21,22,23,24]. In earlier studies, it was established that *Acacia concinna* (Willd.) DC exhibits notable antibacterial and antifungal properties, which can be attributed to the presence of significant secondary metabolites. These metabolites not only showcase the reducing agents and stabilizing agents and their potential for chemotherapeutic applications, but also underscore their medicinal value [22].

In a previous study, a composite of cellulose nanocrystal/silver nanoparticles/waterborne polyurethane [23], cellulose nanofibril/silver nanoparticles composite [1] was investigated, utilizing harmful a chemical reducing agent (NaBH_4_). However, to date, there has been no exploration of simultaneous in situ green synthesis, incorporating *Acacica concinna* pods extracts as a reducing agent in an WPU/CNF/AC-AgNPs composite. The primary objective of this research was to present a facile, economical and time-saving approach for fabricating an antimicrobial composite dispersion technique through the in situ synthesis of AgNPs using green *Acacia concinna* pod extract. In this study, we hypothesized that antimicrobial agent formulations could be produced by incorporating waterborne polyurethane (WPU) and cellulose nanofibril (CNF), along with in situ *Acacia concinna* pods’ crude extract-mediated synthesis of AgNPs. We tested aqueous, 80% ethanol and 80% methanol extracts of *Acacia concinna* pods as potential antimicrobial agents. The formulations were evaluated against Gram-positive bacteria (*S. aureus*) and Gram-negative bacteria (*E. coli*). The WPU/CNF/AC-AgNPs composite dispersion exhibited promising potential as an antimicrobial agent in food packaging systems and other biological-related applications.

## 2. Materials and Methods

### 2.1. Materials

Bionas Sdn. Bhd., Malaysia supplied the crude jatropha oil. Sulphuric acid (H_2_SO_4_) (99%), methanol (CH_3_OH) (99%), formic acid (98%), pyridine (95%) and N-methyl pyrillidone (NMP) (98%) were purchased from Fisher Scientific, Hampton, NH, USA. Triethylamine (TEA) (30%), hydrogen peroxide (H_2_O_2_) (30%), dibutyltin dilaurate (DBTL) (98%), methanol (99.8%), dimethyl propionic acid (DMPA) and acetone (reagent grade) were purchased from R&M chemicals, Tamil Nadu, India. Isophrene diisocyanate (IPDI) (98%) was obtained from Merck, Germany and 1,6-Hexanediol (HDO) was purchased from BDH chemical LTD, London, UK. Ethylenediamine (EDA) (99%) was obtained from Sigma Aldrich (St. Louis, MO, USA). Cellulose nanofiber (CNF) was obtained from Institute of Tropical Forestry and Forest Products (INTROP) (Serdang, Malaysia). Silver Nitrate (99%) (ChemRA, Trier, Germany) and methanol (80%) (R&M chemicals) were purchased from Evergreen Engineering & Resources. All chemicals were reagent grade and were used as received.

### 2.2. Plant Sample Collection and Identification

*Acacia concinna* (Willd.) DC. pods were obtained from Kachin State, and the northern part of Myanmar. The plant samples were taxonomically identified by a botanist, Dr. Khairil Mahmud, and the voucher specimen with the accession number KM 0012/22 was deposited at the Biodiversity Unit, Institute of Bioscience, Universiti Putra Malaysia.

### 2.3. Preparation of AC Pods Extract

For the studies, AC pods were dried naturally in shade at room temperature. Subsequently, the dried fruits and pods were finely powdered using a Waring laboratory blender and then sifted through a 60-mesh sieve to ensure proper refinement. The 60-mesh-sieved AC pods were prepared for extraction using ultrasound-assisted extraction [24]. Initially, AC fine powders were macerated in aqueous, 80% ethanol and 80% methanol into 1 g-to-5 mL ratios at 500 rpm, allowing them to stir (WiseStir MSH-20D hotplate stirrer, Seoul, Republic of Korea) at room temperature overnight. In the second stage, each solution was ultrasonically extracted with a frequency of 20 kHz and a 50 percent amplitude (Sonifier^®^ SFX550, Branson Ultrasonics, Bay City, MI, USA) for 15 min with ice-bath. The filtrate was collected, and the residue was subjected to two additional extractions using the same second stage procedures, renewing the solvents each time. In the final stage, all collected solutions were filtered through a Buchner funnel connected to a vacuum pump. The filtrates were concentrated using a rotary evaporator (Heidolph HB Digital, Schwabach, Germany) at 45 °C to remove the organic reagents. The weight of the extracts was used to determine the yield % and afterwards they were kept for use at 4 °C.

$$\mathrm{Yield}\;\%=\frac{W1}{W2}\cdot 100\%$$

*W*1 is the weight of the extract obtained after drying of solvent.

*W*2 is the weight of the RCM fruit powder.

### 2.4. Preparation of the Waterborne Polyurethane (WPU)

Epoxidised jatropha oil (EJO) was synthesised according to the method reported by [4]. EJO, alcohol, water and sulphuric acid were used to make the jatropha oil-based polyol (JO-P). The calculated amounts of distilled water, CH_3_OH and H_2_SO_4_, (10:9:1) were poured into a beaker and then the mixture was stirred for 15 min at 64 °C. Then, the EJO was added to the mixture, and the reaction continued for 30 min. The mixed compound was transferred to a separating funnel, allowed to cool to room temperature, and the aqueous layer was discarded. After that, excess methanol and distilled water were removed using a rotary evaporator at 60 °C until a clear golden-yellow polyol was produced [25].

The JO-P was added into a neck flask together with DMPA which was previously dissolved in NMP. Mechanical stirrer, torque meter, temperature sensor, nitrogen intake, and dropping funnel were all included in the setting. The mixture was stirred at the rate of 400 rpm for 30 min at 70 °C. This was carried out to form a homogeneous mixture. Then, 1 mL of DBTL was added into the mixture and the reaction was allowed to occur for 30 min. In this reaction, the DBTL acts as a catalyst. Next, IPDI was added drop wise into the mixture for 30 min and the stirring rate was increase to 700 rpm. After 30 min of adding IPDI, the temperature of the reaction was increased to 80 °C. Batches (2 to 5 mL) of acetone were added one at a time. This was carried out to control the viscosity of the mixture. After two hours of additional reaction time, HDO was added. Next, the reactant was allowed to cool down to 35 °C. The process was continued by adding TEA to neutralise the DMPA and disperse it at 1200 rpm with deionised water. Waterborne polyurethane was formed with 38 wt. % solid content. EDA was added and allowed to react for 30 min. The acetone in the mixture was evaporated immediately under vacuum. The prepared WPU was kept as the stock solution for further use [26].

### 2.5. Preparation of WPU/CNF/AC-AgNPs Composite

The preparation of the WPU/CNF/AC-AgNPs composite was carried out according to [27] with slight modification with two steps procedure. In the first step CNF slurry (2.8%, *w*/*v*) was added into 200 mL of 0.1 M silver nitrate (AgNO_3_) aqueous solution. In an ultrasonic bath, the mixture was then exposed to ultrasound waves with a frequency of 20 kHz and a 50 percent amplitude for 30 min. After that, the AC aqueous extract was then carefully added drop by drop to the combined solution as a reducing agent. The mixture was homogenised at 12,000 rpm in a homogenizer (IKA-Labortechnik, Staufen, Germany) until AgNPs were synthesised. The suspension’s colour shifted form pale white to light brown, then dark brown, indicating the synthesis of stabilized CNF/AC-AgNPs.

**Figure 1:**
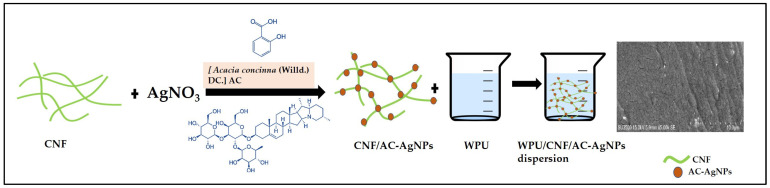
Scheme preparation of WPU/CNF/AC-AgNPs composite dispersion.

In the final stage, a specific amount of CNF/AC-AgNPs suspension (0, 50 mL, 100 mL) was mixed with WPU emulsion (50 mL) and stirred continuously at 1000 rpm overnight at ambient temperature. The samples are denoted as WPU, WPU/CNF/AC-AgNPs-1 and WPU/CNF/AC-AgNPs-2 (Figure 1). All the samples were kept at 4 °C.

### 2.6. Preliminary Screening of Phytochemicals from AC Extracts

The preliminary screening of phytochemicals was performed with the extracted AC pods samples. Table 1 shows the procedure for the qualitative determination of phytochemicals presents in AC pods extract in aqueous, 80% ethanol and 80% methanol solvents.

### 2.7. Disc Diffusion Test on AC Pods Extracts

The disc diffusion technique was used on Muller Hinton Agar (MHA) medium to test the antibacterial activity of extracts. *S. aureus* and *E. coli* were the targets of this test. Before placing the disc paper in a specific spot at the top of the MHA medium, 100 µL of 10^6^ CFU/mL bacteria suspension was dispersed throughout the medium. The 40 µL of AC aqueous, 80% ethanol and 80% methanol extracts were dropped at the centre of each disc. Vancomycin discs (30 µg) and Ampicillin discs (10 µg) were utilised as the positive control, while 10% DMSO served as the negative control. The diameter of the transparent region was measured after 18 h of incubation at 37 °C. All samples, positive and negative controls were carried out three times separately.

### 2.8. UV-Vis Spectrophotometry (UV-Vis)

UV-visible spectrum analysis in the 300–700 nm region was performed on all different formulations of CNF/AC-AgNPs, CNF/AgNO_3_ and AC aqueous extracts (Shimadzu UV-1800 spectrophotometer, Kyoto, Japan). The analysis employed the quartz cuvettes.

### 2.9. Fourier-Transform Infrared Spectroscopy (FTIR)

The functional groups of the samples were analysed using FTIR spectroscopy. The Perkin-Elmer Spectrum 2000 (GmbH, Burladingen, Germany)was employed, which was equipped with a horizontal germanium attenuated total reflectance (ATR). The spectra were obtained with a nominal resolution of 4 cm^−1^ in a range of 400–4000 cm^−1^.

### 2.10. Scanning Electron Microscopy (SEM)

The sample morphologies were examined using a Hitachi SU3500 (SEM; Tokyo, Japan).

### 2.11. Statistical Analysis

All of antimicrobial data were gathered utilising IBM-SPSS version 25, employing one-way analysis of variance (ANOVA). Mean differences were determined using the least significance difference (LSD) method.

## 3. Results and Discussion

### 3.1. Percentage Yield of Extraction and Phytochemical Constituent

The most polar solvents utilized in phytochemical extraction techniques are distilled water, 80% ethanol and 80% methanol. The literature also suggests using these solvents to extract the most bioactive components from plants [32]. The percentage yield and phytochemical composition of aqueous, 80% ethanol and 80% methanol AC pods were shown in Table 2. The yield percentages for AC pods extracted aqueous, 80% ethanol and 80% methanol were 13.2%, 10% and 14% respectively. According to earlier research, the yield percentages of ethanolic extracts of AC using the Soxhlet extractor and maceration were 8.73% and 14.18%, respectively. Moreover, Badi and colleagues discovered that the AC aqueous extract yielded 8.3% through hot maceration [33].The percentage yield of the extract is generally influenced by different types of solvents and extraction methods. Saleh and his colleagues demonstrated a 50% increase in the yield of chlorogenic acid extracted from *Cynara scolymus* L. leaves when using ultrasound-assisted extraction, compared to the conventional maceration process conducted at room temperature [34]. The current percentage yield of the extract shows higher values compared to the previous study due to ultrasonic extraction.

Tannins, phenols, alkaloids, terpenoids and steroids were found in the aqueous, 80% ethanol and 80% methanol solvent extracts of AC. Flavonoids were observed only in the aqueous extracts, while saponins were present only in the aqueous extract of *Acacia concinna* Figure S1. According to research, the preliminary phytochemical analysis includes antioxidant, hormonal, enzyme-stimulating, DNA replication-interfering and antimicrobial effects.

### 3.2. Antimicrobial Activities of AC Extracts

The antimicrobial activities of aqueous, 80% ethanol and 80% methanol ACs were assessed using the disc-diffusion assay. While the aqueous extract of AC demonstrated antibacterial activity specifically against *E. coli* with an inhibition zone of 7.38 ± 0.15 mm, all extracts exhibited antibacterial properties against *S. aureus*, with inhibition zones ranging from 110.51 ± 0.71 to 15.81 ± 0.72 mm, as detailed in Table 3 and Figure S2. The aqueous AC extracts displayed similar antibacterial potentiality to the two positive controls, Vancomycin and Ampicillin. In addition according to [35], some bio-active compound may not be able to fully express their activity when utilizing discs because they may become stuck in the disc’s pores and be unable to move through the inoculation media. Nevertheless, the fact that both extracts show antibacterial activity is noteworthy.

According to S.Todkar and co-authors, the aqueous extract of AC exhibited antibacterial activity, with inhibition zones of 10.4 mm for *B. subtilis* and 5.4 mm for *S. aureus* [36]. It also inhibited *K. pneumoniae*, *P. aeruginoda* and *E. coli*, with inhibition zones measuring 12.5 mm, 5.4 mm and 10.2 mm, respectively. On the other hand, the methanol extract of AC showed antibacterial effects against *B. subtilis* (10.2 mm) and *S. aureus* (6.2 mm), as well as against *K. pneumoniae*, *P. aeruginosa* and *E. coli*, with inhibition zones of 11.4 mm, 5.2 mm, and 10.2 mm, respectively.

Studies on phytochemicals have demonstrated the antimicrobial effectiveness of plants rich in various phytochemicals, including tannins, terpenoids, alkaloids, phenol and flavonoids, which are also described in this study (Table 3) [29]. Additionally, phenolic compounds have a phenol structure, which consists of an aromatic benzene ring and at least one hydroxyl (O-H) group [37]. The disruption of the plasma membrane caused by the accumulation of hydroxyl groups by phenols that interact with membranes is the primary mechanism by which they exert their antibacterial effects. This accumulation modifies the membrane’s hydrophobicity and surface charge, leading to localized ruptures, pore development and leakage, among other disruptive outcomes [38]. According to earlier research on *P. betle* leaf extracts, fatty acids like stearic acid and palmitic acid exhibit antimicrobial properties that are effective against a wide range of infections [39]. Flavonoids are capable of regulating the function of bacterial enzymes that are essential for the survival of the cell, such as those that catalyse the production of cell wall components, cell membrane fatty acid or ATP (adenosine triphosphate) [40].

The results of this study may differ from previous studies regarding the antibacterial effects of AC pod extracts, and this could be attributed to several factors. These factors include the use of different extraction methods, variations in the diffusion capacity of the active substances and differences in the geographic location of the plant materials used in this investigation. Additionally, the use of various organic solvents in this study may have also influenced the antibacterial effects of the AC extract. Thus, it is crucial to take these factors into consideration when interpreting the results of this study and when comparing them to earlier findings. The aqueous AC extract is more active on the candidates compared to 80% ethanol and 80% methanol pod extracts. Therefore, the aqueous AC extracts were further subjected to application as reducing agents in the in situ green synthesis of silver nanoparticles and the crude extract was used for antimicrobial composite dispersion formulation.

### 3.3. UV-Vis Spectrophotometry (UV-Vis)

Growing interest has been generated by recent studies on the production of AgNPs using plant extracts. Numerous plant extracts have been used to encourage the production of AgNPs for various uses. In this study, the potential of the AC pods extract in the green situ synthesis of AgNPs was established for the first time. The plant extract and silver nitrate solution’s colour shift serve as a preliminary indicator which can be used to identify the synthesis of Ag NPs. After incubation, the colourless silver nitrate solution and the light-green fruit extract changed to a dark brown (Ag^+^ to Ag^0^). Silver ions can be drawn to phenolic compounds which have carboxylic and hydroxyl groups. The stability and bio-reduction of the Ag ions are strongly controlled by water-soluble heterocyclic components and polyhydroxylic compounds, respectively. The phenolic compounds in plant extracts may operate as capping agents, exert steric and electrostatic forces and prevent growth around the NPs’ surface [41]. Flavonoids and phenolic compounds may function as capping agents, potentially causing the efficient transformation of Ag^+^ to Ag^0^ [42].

**Figure 2:**
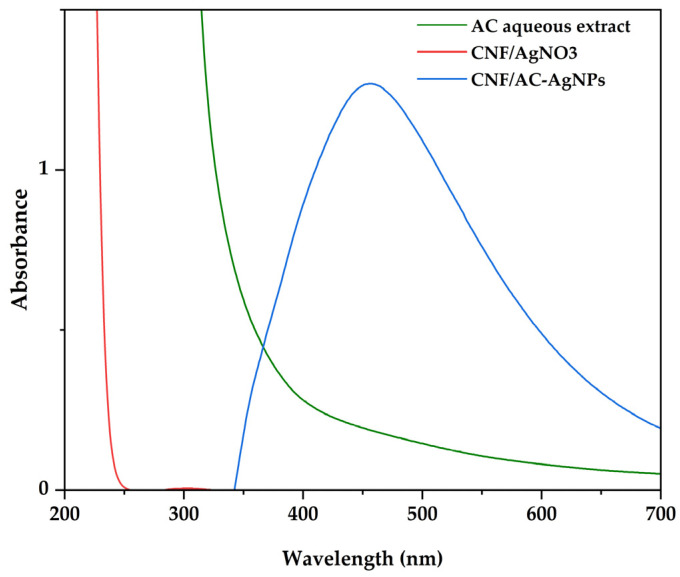
UV-Vis spectra of aqueous AC extract, CNF/AgNO_3_ and CNF/AC-AgNPs.

UV–vis spectrum is one of the most important indicators used to determine the formation of AgNPs in an aqueous solution. Figure 2 presents the absorption spectra of AC-aqueous plant extract, AgNO_3_ and CNF/AC-AgNPs. In this experiment, the silver nanoparticles’ spherical shape and dispersion medium explain the surface plasmon resonance (SPR) which is represented by a band visible at about 450 nm [43]. The resonant oscillation of conducting electrons on the nanoparticle’s surface brought on by an interaction with electromagnetic waves was attributed to the development of SPR. AgNO_3_ and the aqueous AC plant extract did not show absorbance in the visible region of the electrostatic spectrum (400–450 nm). Similar findings were observed in AgNPs synthesised using *Kalanchoe pinnata* leaf extract [44] (Aryan et al., 2021) and *Piper nigrum* seed extract [45] (Kanniah et al., 2021a). According to Lakkin et al., the AgNPs which showed absorption around 440 nm tend to have a spherical shape [46]. *Ocimum tenuiflorum*, *Centella asiatica* and *Clonorchis sinensis* extracts were used to make AgNPs, and the absorbance at 420 nm was measured using a UV spectrophotometer [47].

### 3.4. Fourier Transformed Infrared (FTIR) Spectroscopy

**Figure 3:**
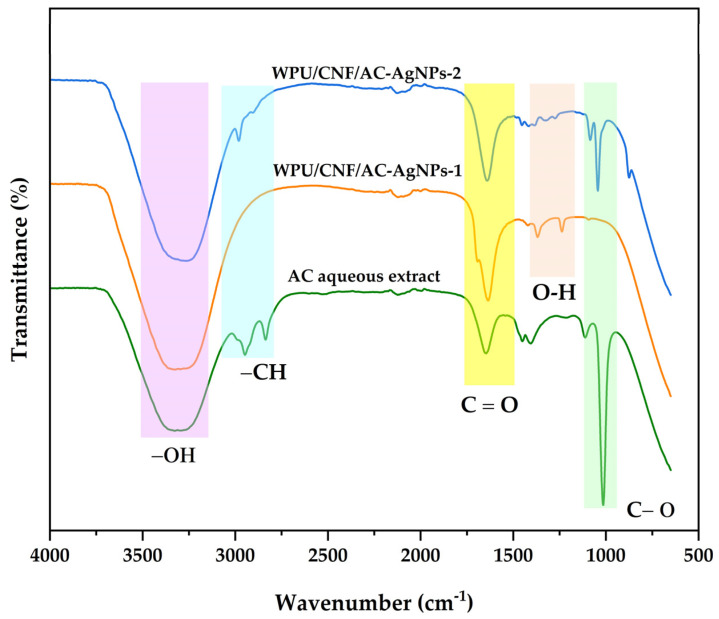
FTIR spectra AC-A, WPU/CNF/AC-AgNPs-1 and WPU/CNF/AC-AgNPs-AC-2.

These samples, which include the aqueous AC extract, WPU/CNF/AC-AgNPs-1 and WPU/CNF/AgNPs-AC-2 are shown in Figure 3 as well as their FTIR spectra. For the aqueous AC extract, the broad peak at 3416 cm^−1^ is assigned to the aromatic OH group. The absorption peak at 2920.36 cm^−1^ is due to SP3 C-H stretch. The absorption peaks at 2860.23 cm^−1^ may be caused by the carboxylic acid group. There is a strong aromatic H-bonded OH stretch at 3428.27 cm^−1^, which increases the likelihood that a phenolic group is present [48]. It was discovered that the high absorption peak between 1699 and 1606 cm^−1^ is caused by the C=O bond. Aldehydes, ketones and carboxylic acids can all be used to indicate the presence of carbonyl compounds. One of the common reducing agents is ketone which functions well when converting metal ions into metal nanoparticles [49]. -C=N stretching and intermolecular/intramolecular hydrogen bonds with medium intensity at 1654 cm^−1^ and O-H stretching and intermolecular/intramolecular hydrogen bonds with medium intensity at 3293 cm^−1^ were observed for the WPU/CNF/AC-AgNPs-1 solution, respectively. Additional -C=O- stretching and –O-H stretching at 1647 cm^−1^ and 3248 cm^−1^ are proof of the presence of carbonyl/carboxylic acid in the sample material. In WPU/CNFs/AC-AgNPs-2, the -C=C- stretching band is located at 1623 cm^−1^ and also at 3280 cm^−1^. In addition, a sharp absorption band between 1021 and 1090 cm^−1^ is assigned to the C=O group of molecules.

According to new research, peaks in the area of 2000–3000 cm^−1^ are mostly attributable to the effects of the atmosphere. Carbohydrates and carboxylic acids have stretching vibrations; thus, they may cause damage. In this instance, C-H and O-H would be present [50]. All of the formulations presented here had this C-H and O-H stretching. In the research carried out in [51], -C=C skeletal vibrations in a phenol ring caused high-frequency peaks between 1350 and 1600 cm^−1^ to occur, whereas the low-frequency peaks between 1050 and 1200 cm^−1^ were caused by the stretching of C-O bonds in the phenols.

### 3.5. Antimicrobial Properties

**Figure 4:**
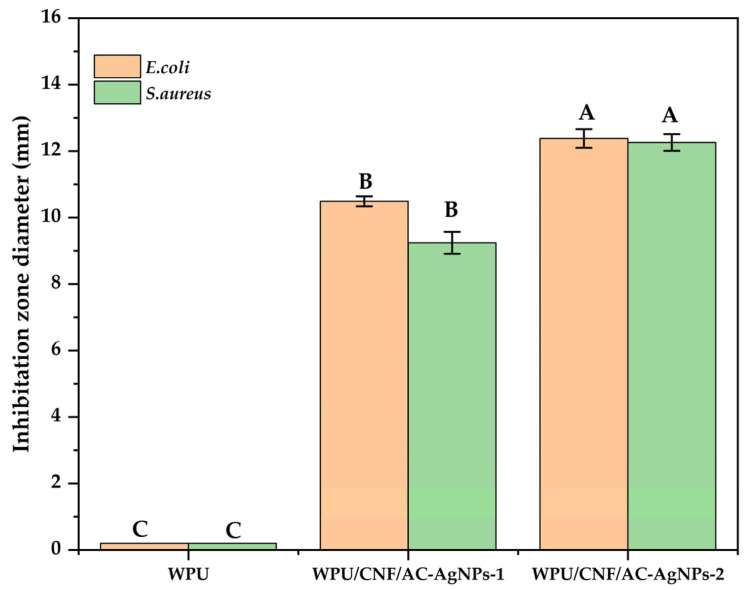
Antimicrobial properties of nanocomposite dispersion. Values reported are the means (*n* = 3) ± SD. Different letters A, B, C on bar graph denote a statistically significant difference between each group (*p* < 0.05).

The disc-diffusion method was used to test the antimicrobial activity of the samples of WPU, WPU/CNFs/AgNPs, WPU/CNFs/AgNPs-AC-1 and WPU/CNFs/AgNPs-AC-2 formulations against bacteria, including a strain of the Gram-negative bacteria *E. coli* and a strain of the Gram-positive bacteria *S. aureus*, as can be seen in Figure 4 and Figure S3.The diameter of the disk was 6 mm. WPU alone did not show any antimicrobial activity while WPU/CNF AgNPs showed activity against *E. coli* with an inhibition zone of 12.27 ± 0.5 and against *S. aureus* with an inhibition zone of 12.30 ± 0.19 mm. The antibacterial properties of silver nanoparticles are linked to the release of Ag+ ions, which bind to the abundantly present negatively charged functional groups in membrane proteins such sulfhydryl, carbonyl, imidazole, amino and phosphate to change the structure of the membrane and enhance its permeability. Ag^+^ ions disrupt the cellular transport mechanism, which results in cell death [34,35]. For WPU/CNFs/AgNPs-AC-1, the inhibition zones were 7.29 ± 0.4 mm for *E. coli* and 6.38 ± 0.052 mm for *S. aureus*. As WPU/CNFs/AgNPs-AC-2, the inhibition zone for *E. coli* was 10.63 ± 0.3 mm, whereas the inhibition zone for *S. aureus* was 9.62 ± 0.32 mm. In the research in [36], they stated that the inhibition zones (mm) for *E. coli* and *S. aureus* were 7.8 and 6.2 mm. They also stated that the outcome of their investigation demonstrates that the extracts of plants used displayed possible antimicrobial activity against the tested pathogens. The current investigation supports the idea that numerous medicinal herbs could be beneficial as antimicrobial agents. In this investigation we utilised solely crude extracts of pods from *Acacia concinna*. The study involved the profiling of secondary metabolites that have been demonstrated to have antibacterial action in terms of inhibiting and blocking crucial enzymes needed for the development and metabolism of microbes [22].

It should be noted that the inhibition zones of the antimicrobials against the bacterial colonoes do not develop in a circular radius surrounding the antimicrobial spot. According to new research, the addition of a CNF/AgNP composite (1–10 mg/mL) resulted in a declining trend in the number of *E. coli* O157:H7 in the first 6 h, and the inhibitory effects were concentration-dependent in the second 6 h. Antimicrobials may be activated utilising CNF/AgNP since then. The results of the investigation into the antimicrobial activities of these plant extracts were presented in Figure 3.

### 3.6. Morphology of WPU/CNF/AC-AgNPs Composite Films

**Figure 5:**
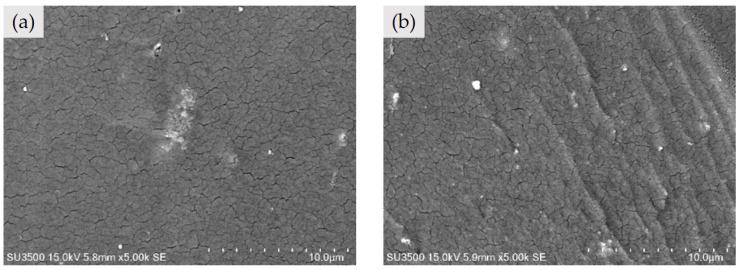
SEM images of WPU/CNF/AC-AgNPs-1 (**a**), WPU/CNF/AC-AgNPs-2 (**b**).

Scanning Electron Microscopy (SEM) images of WPU/CNF/AC-AgNPs-1 and WPU/CNF/AC-AgNPs-2 are shown in Figure 5a and Figure 5b, respectively. The films showed a flat surface and uniform colour, indicating the homogeneous dispersion of the WPU, CNF and AgNPs with some cracks and aggregates. For the WPU/CNF/AC-AgNPs-1 composite, aggregated white particles were observed on the surface. This suggests that individual silver nanoparticles were present on the CNFs’ surface, and the WPUs may cluster together as cellulose fibrils intertwine during film preparation [1]. In addition, a rough and wrinkled surface can be observed in the film. Moreover, increasing the content of CNF/AC-AgNPs in the WPU matrix led to a relatively rougher surface. This aggregation likely results from an excess of nanoscale filler being introduced into the mixed matrix.

## 4. Conclusions

Here, an innovative, simple, cost-effective and environmentally friendly method for the fabrication of WPU/CNFs/AgNPs through ultrasound-assisted green in situ synthesis of silver nanoparticles into WPU and CNF was successfully demonstrated. It is worth noting that the aqueous AC pods extract exhibited superior reduction and clapping abilities for the stabilization of the silver nanoparticles compared to the ethanol and methanol extracts of AC pods. Furthermore, the AC pods extracts demonstrated robust antimicrobial properties against both Gram-positive and Gram-negative bacteria. The WPU/CNFs/AgNPs-AC composite dispersion identified as a potentially effective antimicrobial agent can be utilized as a natural alternative to chemical antibacterial agents in various applications, ranging from biological coatings to the food industry.

## Figures and Tables

**Table 1:** Preliminary screening of phytochemicals.

No	Phytochemical Name	Method	Observation	References
1.	Phenols	Sample + 5 drops of 2% (*w/v*) FeCl_3_	Black or Blue-green colouration	[28]
2.	Flavonoid	Sample + 5 drops diluted NaOH + diluted HCL	Yellow solution with NaOH turned colourless with HCL	[29]
3.	Alkaloid	Sample + 5 drops of Dragendroff’s reagent	The orange colour was formed	[29]
4.	Tannins	Sample + 2 mL 2% FeCL_3_	Blue-green or black colouration	[30]
5.	Terpenoid	Sample + 3 mL chloroform+ 2 mL Con.H_2_SO_4_	Reddish-brown colour	[29]
6.	Saponin	Sample + 4 mL distilled water (shaken)	Foam formation	[29]
7.	Steroids	Sample + 4 drops Chloroform + Conc H_2_SO_4_	Red colour in the chloroform layer	[31]

**Table 2:** Phytochemicals screening and percentage yield of AC extracts.

	Aqueous Extract	80% Ethanol Extract	80% Methanol Extract
Yield %	13.2%	10%	14%
Phytochemical			
Phenols	+	+	+
Flavonoid	+	-	-
Alkaloid	+	+	+
Tannins	+	+	+
Terpenoids	-	+	+
Saponins	+	-	-
Steroids	+	+	+

+ = Presence; - =Absence.

**Table 3:** The antibacterial activities of aqueous, 80% ethanol and 80% methanol AC extracts.

Sample	Zone of Inhabitation	
	*E. coli* (mm)	*S. aureus* (mm)
Aqueous	7.38 ± 0.15 ^B^	15.81 ± 0.72 ^B^
80% ethanol	-	10.51 ± 0.71 ^D^
80% methanol	-	13.2 ± 0.18 ^C^
Vancomycin (30 µg)	7.55 ± 0.23 ^B^	19.94 ± 0.57 ^A^
Ampicillin (10 µg)	11.15 ± 0.12 ^A^	6.85 ± 0.05 ^E^
DMSO (10%)	0	0 ^F^

Values are means ± SD of three determinations. Means within each column with different letters differ significantly (*p* < 0.05).

## Data Availability

Data are contained within the article.

## Supplementary Materials

The following supporting information can be downloaded at: https://www.mdpi.com/article/10.3390/polym16192683/s1, Figure S1 phytochemical screening of AC aqueous, ethanol and methanol extract; Figure S2 of antimicrobial properties of AC pods extracts; Figure S3 of antimicrobial properties of WPU/CNFs/AgNPs-AC composite dispersion.
