# Supplementary material for: Antimicrobial Effect of Waterborne Polyurethane-Based Cellulose Nanofibril/Silver Nanoparticles Composites and Acacia concinna (Willd.) DC Extract (Shikakai)
Source: Polymers (Basel). 2024 Sep 24;16(19):2683. doi: 10.3390/polym16192683 (PMC11478976; doi:10.3390/polym16192683)
Supplement: Supplementary file 1 [file polymers-16-02683-s001.zip › polymers-2826628-supplementary.pdf]

Antimicrobial effect of waterborne polyurethane-based cellulose nanofibril/silver nanoparticles composites and Acacia concinna extract (shikakai)

Lu Lu Taung Mai [1, 2], H'ng Paik San [1, 3*], Min Min Aung [1*], Hiroshi Uyama [4,] Ainun Zuriyati Mohamed [1,] Ezyana Kamal Bahrin [5], Mas Jaffri Masarudin [6], Azra Afrina binti Mohamad Zulkifli[7], Tung Woey Chew [1]

[1]Higher Education Centre of Excellence (HiCoE), Institute of Tropical Forestry and Forest Products (INTROP), University Putra Malaysia, 43400 UPM Serdang, Selangor, Malaysia.

[2]Department of Chemistry, University of Myitkyina, 01011, Myitkyina, Kachin State, Myanmar

[3]Department of Forestry and Environment, Faculty of Forestry, Universiti Putra Malaysia, 43400 UPM Serdang, Selangor, Malaysia.

[4]Department of Applied Chemistry, Graduate School of Engineering, Osaka University, 2-1 Yamadaoka, Suita, Osaka 565-0871, Japan.

[5]Department of Bioprocess Technology, Faculty of Biotechnology and Biomolecular Sciences, Universiti Putra Malaysia, 43400 UPM Serdang, Selangor, Malaysia

[6]Department of Cell and Molecular Biology, Faculty of Biotechnology and Biomolecular Science, Universiti Putra Malaysia, Serdang 43400, Malaysia

[7]Department of Chemistry, Faculty of Science and Technology, Universiti Putra Malaysia, 43400 Serdang, Selangor, Malaysia

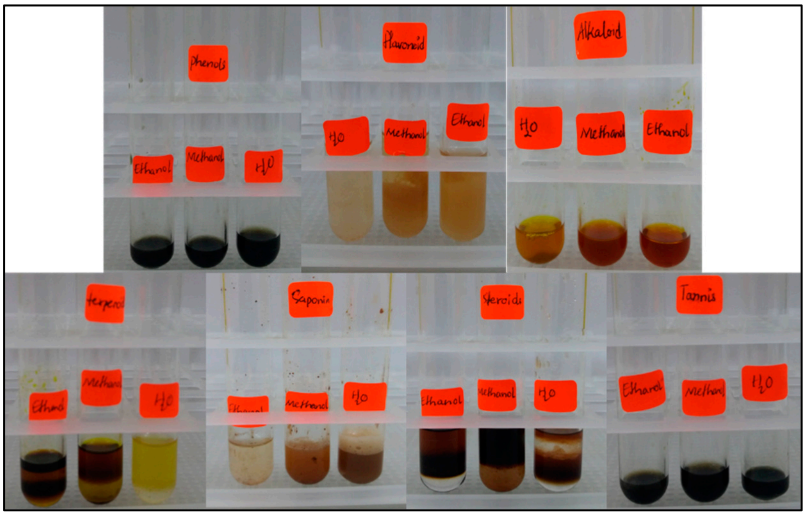

Figure S1 Phytochemicals screening of aqueous, ethanol and methanol extracts of *Acacia concinna (Willd.)* DC. pods.

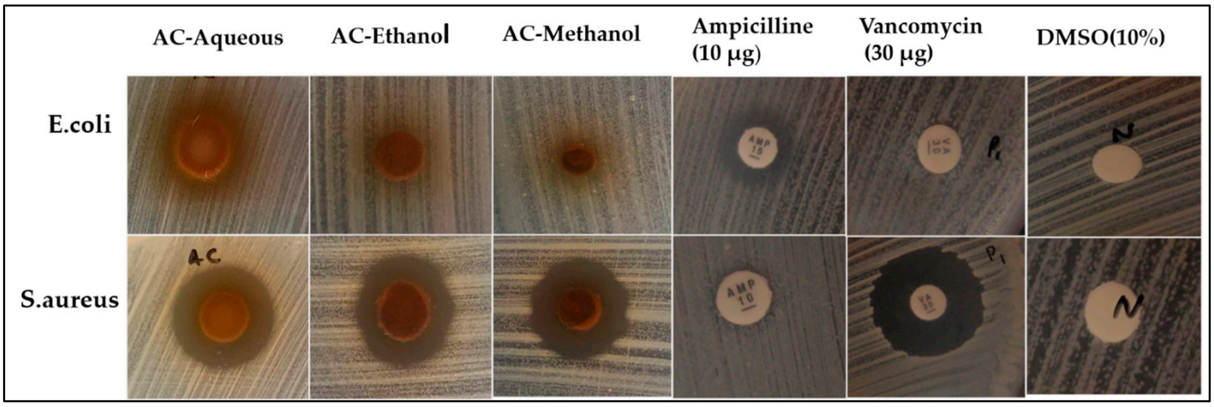

Figure S2 Antimicrobial activity of AC extracts.

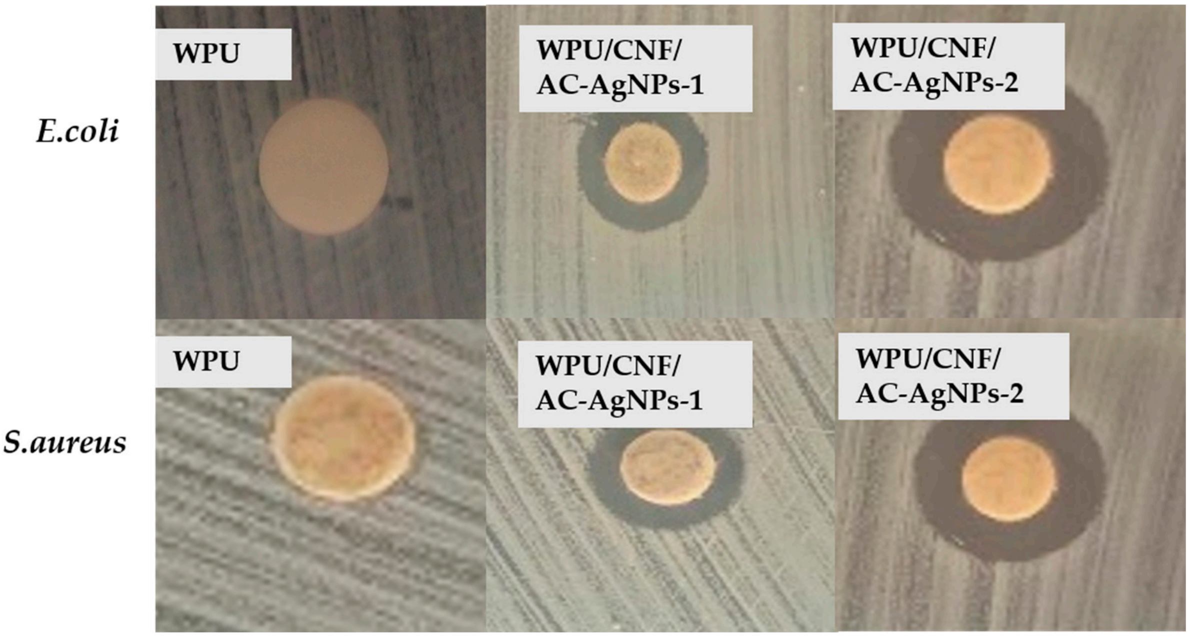

Figure S3 Antimicrobial activity of WPU/CNF/AC-AgNPs composite dispersion.
